# Pulmonary Artery Catheter Seemingly Entrapped in the Skull

**DOI:** 10.1155/2019/9842129

**Published:** 2019-08-05

**Authors:** S. M. Friedman, G. R. Rajan

**Affiliations:** ^1^UC Irvine School of Medicine, Irvine, USA; ^2^Department of Anesthesiology & Perioperative Care, UC Irvine School of Medicine, Irvine, USA

## Abstract

A pulmonary artery catheter is an important tool for the monitoring of hemodynamics in patients. Unfortunately, misplacement of a catheter tip may occur in the vasculature local to the intended placement. Misplacement of the catheter can be further complicated by entrapment at the unintended destination. We present a case of a misplaced and entrapped pulmonary artery catheter in a patient with worsening pulmonary disease. After multiple unsuccessful attempts to float the catheter, it was partially retracted and found to be stuck. Imaging showed the tip terminating in the right internal jugular vein at the level of the jugular foramen. It was initially suspected that the catheter had become looped, knotted, or otherwise entangled within the vasculature of the skull and surgical removal would be necessary. Before surgical removal was performed, it was instead determined that the catheter had become kinked and entrapped at the end of the introducer sheath, and noninvasive removal was accomplished by first removing the introducer sheath.

## 1. Introduction

Over the last several decades, the placement of a pulmonary artery catheter has become an important tool for the monitoring of cardiac and pulmonary hemodynamic variables. However, as with any procedure, there are many possible complications, including venous or arterial misplacement [1]. In most instances, improper placement alone is not a serious complication, but on some occasions, it can be further complicated by entrapment most commonly due to looping, knotting, or catching on a foreign body [2, 3]. In cases of entrapment, a variety of methods exist for retrieval. Radiologically guided snares or even surgical removal are often employed, as prolonged manipulation may cause injury to the cardiac or vascular lining or provoke cardiac arrhythmia [3]. Here we describe a case of a misplaced pulmonary artery catheter with the tip seemingly entrapped at the junction of the right internal jugular vein and sigmoid sinus intracranially, as well as our successful noninvasive removal of the catheter.

## 2. Case Description

A 66-year-old male (height 5'2”, weight 50.9 kg) with a history of severe idiopathic pulmonary fibrosis with two spontaneous pneumothoraces and a persistent bronchopleural fistula underwent a successful leak repair via pleurodesis. Following the procedure, the patient developed acute respiratory failure with persistent hypoxia requiring intubation and mechanical ventilation. An echocardiogram showed right ventricular strain with an EF of 78%, right ventricular systolic pressure of 115 mmHg, and enlarged pulmonary arteries, suggestive of obstructive shock from a pulmonary embolism or pulmonary hypertension. Limited improvement prompted the need for monitoring beyond that provided with transesophageal echocardiogram. Initiation of a right heart catheterization with a pulmonary artery catheter allowed continuous real-time monitoring of hemodynamics and assessment of pulmonary capillary wedge pressure, to further guide therapy towards a potential lung transplant.

A critical care fellow, under the supervision of an attending physician, used an ultrasound-guided technique to successfully place a 9 Fr introducer sheath [Arrow International, PA, USA] in the right internal jugular vein, through which the pulmonary artery catheter [Edwards Lifesciences, CA, USA] was inserted. During the initial attempt to float the catheter, right atrial, and ventricular wave forms were confirmed with the catheter advanced 20-30 cm, and a pulmonary artery wave form was confirmed at about 40-50 cm. Pulmonary artery pressures were measured varying between 100/30 mmHg, but wedging of the catheter was unsuccessful. Subsequent attempts confirmed appropriate right atrial and ventricular wave forms but failed to advance through the pulmonary artery. During the final attempt, the catheter was advanced to about 40-50 cm without acquiring a pulmonary artery wave form, but upon attempted removal, it stopped at approximately 30 cm, without any possible further retraction. The inability to remove the catheter prompted the use of imaging to evaluate catheter misplacement due to possible congenital anomalous anatomy or intravascular entrapment.

**Figure 1:**
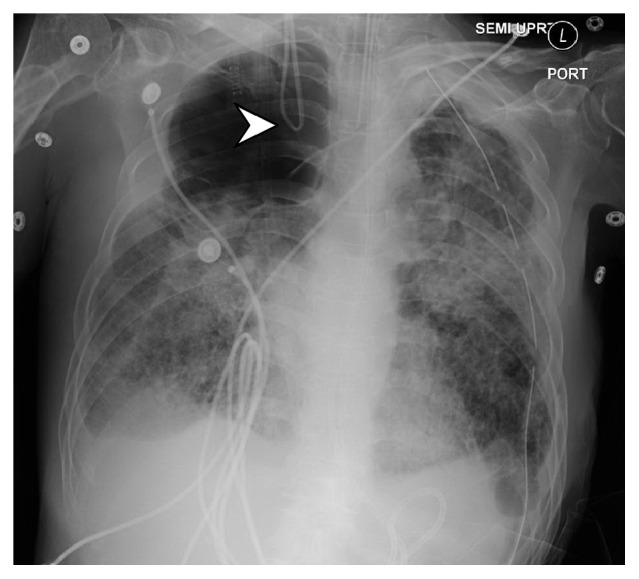
Portable chest x-ray with arrow depicting retrograde looping of pulmonary artery catheter.

**Figure 2:**
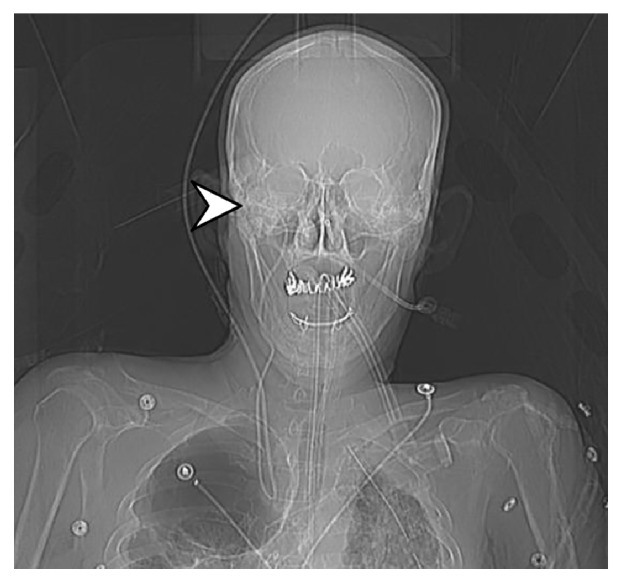
CT Venogram of the head with arrow depicting mispositioned tip of the venous catheter within the right internal jugular vein at the level of the jugular foramen.

Portable chest x-ray showed the catheter with complete retrograde looping directed upwards off the film (Figure 1). A further CT Venogram of the head showed the tip of the catheter terminating within the right internal jugular vein at the level of the jugular foramen (Figure 2). At this point, it was thought that the catheter had become entrapped via looping or knotting within the vasculature of the skull, and the neurosurgical team was consulted that determined that removal would likely require a surgical procedure involving incision of the jugular vein.

During the preoperative evaluation of the patient, however, it was determined that the catheter might be stuck at the tip of the introducer sheath, and it might be possible to remove the catheter first by carefully withdrawing the introducer sheath. The catheter was then removed under aseptic conditions by first withdrawing the introducer sheath over the pulmonary artery catheter. The catheter was then gently pulled in the cephalad direction followed by the caudal direction. All through the procedure, the balloon remained completely deflated and utmost caution was exercised to apply only the minimal traction necessary. Given this eventual solution, it is likely that in the initial attempt to retract the catheter, the proximal part of the loop was the first retrieved, diminishing the size of the loop with limited movement of the tip. This caused the formation of a tight kink at the exit point of the introducer sheath, thus entrapping the catheter.

## 3. Discussion

The pulmonary artery catheter remains an effective method for monitoring hemodynamic variables, as well as administering medications such as inotropes, in the management of complex cases. As they continue to be used, it is important to remember that their use is not without potential complications such as improper placement. Likely the most dangerous misplacement involves the accidental puncture of an artery during initial access, which, if unrecognized, can lead to the arterial placement of a catheter in the carotid or subclavian arteries [1]. Somewhat less dangerous arterial misplacement may sometimes be possible by the passage of the catheter through a cardiac septal defect [4]. In contrast, venous misplacement can occur via the looping of pulmonary artery catheter as it is advanced, such as in the case we have presented. In some instances, this can lead to the tip of the catheter terminating in the vena cava, intrahepatically, or as we have shown here, within the skull [2, 3]. In many of these situations, misplacement alone is not a serious complication, as the catheter can usually be withdrawn and placed appropriately.

In some cases, however, improper placement may be further complicated by the entrapment of the catheter within the vasculature. This may be possible through the aforementioned looping of the catheter, which can lead to the formation of one or, in some cases, even multiple knots which prevent retrieval [4, 5]. Pulmonary artery catheters have also become caught on other foreign bodies such as a surgical suture or automatic implantable cardioverter-defibrillator coil [2, 6, 7]. Regardless of the cause, an entangled catheter may put the physician in need of an unconventional solution.

A variety of methods for retrieving an entrapped catheter exist, but it is important to take care in minimizing the manipulation of the catheter, as prolonged manipulation can cause injury to the cardiac or vascular lining or provoke cardiac arrhythmia [3]. One common method for retrieval is through the use of interventional radiological techniques involving a variety of snares such as single-loop, triple-loop, or entwined guidewire techniques [8]. If this is unsuccessful, or impractical due to positioning, it may even be necessary for surgical removal of a knotted or stuck catheter [9]. Here we have described a case where the tip was seemingly entrapped in an unfortunate location, for which a surgical removal was nearly performed.

We believe our case describes a novel mechanism of entrapment of a pulmonary artery catheter by kinking at the distal tip of the cordis, which appeared via imaging to be stuck within the skull. Further, the report details a step-by-step explanation of how to disentangle and remove the catheter through noninvasive intervention. In the future, utmost care in monitoring of pressure readings on insertion and avoidance of overextension of the catheter may prevent similar situations from occurring altogether. In the unfortunate event of an entrapped catheter, where imaging shows a tight bend near the exit from the introducer sheath, we hope this report may guide the removal of the catheter by first carefully withdrawing the introducer sheath.
